# ¿Sabes si viene un code-switch? Bilinguals can predict upcoming code-switches given enough context

**DOI:** 10.3758/s13421-026-01877-3

**Published:** 2026-03-30

**Authors:** Lauren K. Salig, Jorge R. Valdés Kroff, Jeymi Menendez, Mia C. Lulli, L. Robert Slevc

**Affiliations:** 1 Program in Neuroscience and Cognitive Science, University of Maryland, College Park, MD USA; 2 Department of Psychology, University of Michigan, Ann Arbor, MI USA; 3 Department of Spanish and Portuguese Studies, University of Florida, Gainesville, FL USA; 4 Department of Psychology, University of Maryland, College Park, MD USA

**Keywords:** Prediction, Bilingualism, Code-switching, Comprehension

## Abstract

Language comprehension involves prediction, but what exactly can comprehenders predict? We ask if bilinguals can consciously predict upcoming code-switches between languages. If predictions focus on a sentence’s message, then listeners should not predict code-switches, which alter the language used but not necessarily the meaning. Alternatively, if listeners predict at many levels—including language—then they may predict code-switches. Here, we selected sentences from a corpus of spontaneous bilingual conversations that started in Spanish and removed the final word that would have continued in Spanish or code-switched to English. In two preregistered experiments, Spanish–English bilinguals heard these sentence fragments and predicted the language of the omitted final word. They successfully predicted upcoming code-switches when given 30 seconds of context prior to their predictions (Experiment 1; *N* = 94), but not when only hearing the sentence fragment (Experiment 2, *N* = 115). This shows that bilinguals can predict upcoming code-switches when sufficient conversational cues are available.

## Introduction

Can you guess what word comes ____? Speech is fast and fleeting, yet listeners understand it without much trouble—in part due to the predictive processes that allowed you to fill in the blank with the word “next.” Comprehenders predict many aspects of upcoming speech (at least sometimes; Brothers et al., 2017). Successful predictions aid comprehension (Boudewyn et al., 2015), and incorrect predictions hinder comprehension (Bannon et al., 2024) while improving future predictions via error-based learning (Dell & Chang, 2014).

But what happens when comprehending speech that suddenly switches into another language? Such code-switching is common among bilinguals and follows certain patterns (e.g., Gardner-Chloros, 2009). On one hand, given that code-switches do not occur randomly and that bilingual communication seems unimpaired by code-switches (Gardner-Chloros, 2009), one might imagine that bilinguals also predict code-switches during comprehension (Valdés Kroff & Dussias, 2023). This fits with the broader idea that prediction occurs at multiple levels of linguistic representation (Heilbron et al., 2022).

On the other hand, prediction in language is often assumed to reflect an attempt to infer the *message* being communicated (Kuperberg & Jaeger, 2016). While code-switches carry social/pragmatic information (Gumperz, 1982), they do not necessarily impact the conceptual message (e.g., "Mira el sky" has the same literal meaning as "Look at the sky"). Thus, the specific language might not be a target of prediction. Some evidence that comprehenders may not effectively anticipate and prepare for upcoming code-switches comes from findings of “switch costs,” when bilinguals take longer to process—or demonstrate brain responses that indicate difficulty processing—code-switched sentences compared to single-language equivalents (e.g., Altarriba et al., 1996; Bultena et al., 2015). Thus, encountering code-switches seems to present difficulties relative to single-language comprehension, just as unpredicted information can impede comprehension (Bannon et al., 2024).

Switch costs are often found in psycholinguistic studies, but not always (Gullifer et al., 2013), especially when considering code-switches in their typical contexts (ecologically valid switches). The types of code-switches that occur more frequently in natural production generally elicit little-to-no switch costs in comprehension; for example, Spanish–English bilinguals process more common noun code-switches (e.g., *el soap* “the soap”) with more ease than less common verb code-switches (e.g., *han cooked* “have cooked”; Salig et al., 2024). Further, switch costs are generally smaller for those with more code-switching experience and when switches occur in a more natural context (Beatty-Martínez & Dussias, 2017; Kaan et al., 2020). These findings suggest that code-switches in typical experiments, which often present decontextualized, isolated sentences, might be relatively *un*predictable compared to more naturalistic code-switches. When switches are ecologically valid and thus, in principle, predictable, they seem to elicit relatively little processing cost. Here, we ask if this could reflect bilinguals’ ability to predict code-switches in ecologically valid input.

### How might comprehenders predict code-switches?

In ecologically valid contexts, comprehenders have information that may allow them to predict code-switches. For one, anticipatory articulation changes can happen before a switch occurs; for example, the shortening of voice onset times (VOT) before switching from English to Spanish and the slowing of local speech rate prior to a code-switch (Balukas & Koops, 2015; Fricke et al., 2016; Piccinini & Arvaniti, 2015). Additionally, the phonotactic constraints for each language may point towards upcoming switches (e.g., one language may disallow consonant clusters in onsets; P. Li, 1996). Indeed, the presence of such cues can facilitate comprehension of code-switched sentences (Fricke et al., 2016).

There are also nonarticulatory cues to upcoming, ecologically valid code-switches. Code-switches often occur at points of speech-planning difficulty (perhaps strategically; Johns & Steuck, 2021). Thus, code-switches tend to co-occur with lower-frequency words, surprising content, and disfluencies (Fricke et al., 2016; Hlavac, 2011; Myslín & Levy, 2015). Further, there are lexical and syntactic regularities associated with code-switches. Code-switches are more frequent near cognates and after other code-switches (Broersma et al., 2020; Kootstra et al., 2020). Additionally, code-switches are more likely at certain syntactic junctures (e.g., within noun phrases over verb phrases), and listeners seem able to track these patterns (Beatty-Martínez & Dussias, 2017; Guzzardo Tamargo et al., 2016; Salig et al., 2024).

More broadly, a listener may know their conversational partner’s propensity to code-switch based on speaker familiarity or by extrapolating from characteristics like age (e.g., Myslín & Levy, 2015). Physical location and conversational topic may also inform expectations for code-switching, as certain words may be favored in a particular language based on lexical access, social/cultural associations, or lexical alignment established throughout a conversation (Aaron, 2015; Li et al., 2024; Sarkis & Montag, 2021). Perhaps even facial or bodily expressions could indicate if a speaker may subsequently code-switch.

Listeners may use any combination of these cues to predict code-switches, and their ability to predict may depend on individual factors such as code-switching experience. However, the existence of these cues—or even facilitated processing of code-switches given such cues (Fricke et al., 2016)—does not necessarily indicate that bilinguals can *predict* code-switches. Instead, such findings might reflect easier integration of (yet unpredicted) code-switches given contextual support.

### The current study

**Fig. 1:**
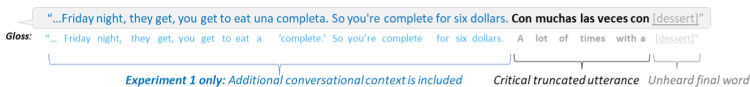
Study design. This figure shows an example code-switch condition trial. In Experiment 1, participants heard 30 s of audio (which sometimes, but not always, included code-switches) before it was cut off before the final (unheard) word. Here, some of that additional context is shown in blue text (the additional context for this example item was actually longer and has been reduced here for space). In Experiment 2, only the critical truncated phrase was heard by participants, again with the final word being unheard. (Color figure online)

In two experiments, we investigated whether bilinguals can capitalize on naturally present cues to predict code-switches in ecologically valid sentences. Spanish–English bilinguals heard utterances from the Bangor-Miami corpus (Deuchar et al., 2014) of natural bilingual speech that started in Spanish and were truncated before the final word. Participants explicitly predicted whether the final word would continue in Spanish or code-switch to English. They either heard 30 seconds of conversation leading up to the point where they made their prediction (Experiment 1) or heard only a single sentence fragment (Experiment 2; see Fig. 1). Since the sentences were naturalistic speech, presumably containing multiple cues to upcoming code-switches, we predicted that bilinguals could discriminate to-code-switch sentences from sentences that would remain in a single language. We further hypothesized that individuals with more code-switching experience would be better able to predict code-switches and that each conversation’s characteristics may affect prediction success.

To anticipate the findings below, comprehenders could, in fact, successfully predict code-switches, but only for fragments embedded in a larger context. This informs our understanding of bilingual comprehension and linguistic prediction more generally, while raising questions for future study about exactly how and when such predictions occur. These findings also have methodological implications, given that comprehension in isolated sentence fragments (akin to common experimental designs) differed compared with comprehension in relatively longer and more natural contexts.

## Experiment 1

Both experiments were preregistered (Experiment 1: https://osf.io/hf83m/; Experiment 2: https://osf.io/29uyc/). Analyses were conducted according to the preregistrations, except where indicated as exploratory. Materials, data, and analysis scripts are available on the Open Science Framework (OSF; https://osf.io/q9spv/). Participants in both experiments completed informed consent before participating.

### Method

#### **Participants**

We recruited 104 US-based, Spanish–English bilinguals through Prolific (*N* = 50, https://www.prolific.com/) and the University participant pool (*N* = 54), aiming for 100 include-able participants to achieve reasonable power for detecting a main effect in a within-subjects bilingualism study (Brysbaert, 2021). Participants were at least 16 years old, self-reported normal hearing, and indicated that they speak Spanish and English. We excluded ten participants based on the preregistered exclusion criteria of not indicating Spanish and/or English as their first and most-preferred language and/or not passing at least two of the three attention checks (see below).

The analysis included 94 bilinguals (54 women, 37 men, three another gender). The bilinguals had varied language experiences (see Table 1), but generally learned both languages from a young age, showed proficiency in both languages, and had more daily exposure to English (*M* = 67.70%) than Spanish (*M* = 30.77%) with minimal other-language exposure (*M* = 1.53%). Most participants preferred using English (*N* = 64), although some preferred Spanish (*N* = 3), and many had no preference (*N* = 27). Two participants reported never code-switching, but those who did code-switch started at a young age (*M* = 7.66 years, *SD* = 5.00).

**Table 1:** Participant characteristics

	Experiment 1	Experiment 2
Analyzed Sample Size (*N*)	94	115
Age (years)	28.28 (12.98)	30.12 (10.30)
AoA English (years)	1.72 (2.26)	1.72 (2.51)
AoA Spanish (years)	3.95 (8.48)	3.72 (5.84)
English LexTALE score (out of 100)	86.36 (12.13)	90.13 (9.46)
Spanish LexTALE score (out of 100)	59.29 (12.34)	62.11 (10.46)
Exposure Entropy (from 0 to 1.58)	0.87 (0.24)	0.81 (0.25)
BCSP score (from 0 to 100)	47.72 (14.42)	47.09 (15.49)

Table shows mean value with standard deviation in parentheses, except for sample size. AoA = Age of Acquisition. English vs. Spanish LexTALE scores are not directly comparable, and LexTALE assessments may underestimate verbal proficiency. Higher exposure entropy indicates more balanced daily exposure to Spanish, English, and other languages. BCSP = Bilingual Code-Switching Profile (higher indicates more code-switching experience)

#### **Materials**

Both experiments’ materials were selected from the Bangor-Miami corpus (Deuchar et al., 2014), a spoken language corpus in which Spanish–English bilinguals of Hispanic heritage recorded spontaneous conversations with their typical conversation partners. Participants were told that researchers were interested in how bilinguals communicate with no specific reference to code-switching, and no researchers were present during recordings, resulting in informal, naturalistic conversations with ample turns in English, Spanish, and with code-switches.

We focus on Spanish-to-English code-switches because this is the most common reported switch direction for Spanish–English bilinguals in Miami, which is reflected in the Bangor-Miami corpus (Fricke & Koostra, 2016, see also Tomić & Valdés Kroff, 2022, for expanded discussion on this design choice).

#### **Item creation and matching**

We selected 30 Spanish phrases with a final word that code-switched into English and matched them with 30 Spanish phrases with a final word in Spanish. Final words were nouns. Code-switched and matched items did not differ in utterance length (both averaging 7.83 words) or final word frequency (averaging 46.0 and 46.2 per million words, respectively, but note that participants never heard the final word; |*t*|s ≤ 0.01, |*p*|s ≥.99). The word prior to the noun came from a number of syntactic categories, with the four most common categories being determiners (16), prepositions (5), possessives (3), and adjectives (3) for code-switched phrases and determiners (19), verbs (5), prepositions (2), and adjectives (2) for Spanish-only phrases. There was a 2:1 female-to-male speaker ratio for the phrases in each condition.

To create the audio files, we removed each phrase’s final word, then cut the files to exactly 30 s (with a short fade-in at the beginning), so participants heard some ecologically valid conversation preceding the critical phrase and the final phrase itself (minus the final word). For Experiment 2, audio files were cut to include only the final critical utterance (minus the final word), which averaged 2.35 s (*SD* = 0.73 s).^1^

For Experiment 1, the context portion of the audio preceding the final phrase was not controlled between the two conditions in favor of prioritizing ecological validity. That is, the conversational contexts preceding code-switches potentially have differences from their matched counterparts, but these differences likely reflect real-world variability that listeners may capitalize on (see Conversational Context Factors in Prediction Success section below for more on differences between the two conditions).

#### **Procedure and design**

This was a factorial within-subjects design in which every participant heard all audio files—both the audio files that ended in phrases that (in their pretruncated versions) code-switched to English and those that stayed in Spanish.

Participants completed the study remotely on their own device. The experimental task was administered using PCIbex (Zehr & Schwarz, 2018). They completed a task to determine if they were wearing headphones; if they failed, they were asked to put on headphones before the study began (Woods et al., 2017). The instructions explained the experimental task in which participants predicted the language of the final unheard word of each audio file—in other words, if there would be a code-switch. Participants were requested to respond quickly, going with their first instinct, although the task was not timed. The instructions included two examples (final phrase only) in which participants heard the full phrase (including the final word) and then a version with the final word removed. Then, they completed two longer-length practice trials with feedback before beginning the experiment. At that point, participants were asked to predict how well they would perform on the task on a 0–99 scale.

In each trial, participants clicked "Play" to hear the 30 s of conversation that immediately preceded the unheard final word (see Fig. 1). An audio icon was presented while the audio played. Then, participants chose the language they believed would come next in the sentence, given six options: Definitely English, Probably English, Maybe English, Maybe Spanish, Probably Spanish, or Definitely Spanish. “English” selections indicated a code-switch prediction, while “Spanish” selections indicated a no-switch prediction. Participants could not replay the audio and did not receive feedback.

In addition to critical trials, there were 3 attention checks. Instead of hearing a corpus audio file, participants heard an audio file that explicitly told them which response to select for that trial. The order of all trials was randomized for each participant.

After the experimental task, participants provided a self-evaluation of their performance on a 0–99 scale. Then, participants completed the English and Spanish LexTALE vocabulary assessments (Izura et al., 2014; Lemhöfer & Broersma, 2012) with order counterbalanced across participants. Finally, participants completed a language background and demographic survey, and the Bilingual Code-Switching Profile (BCSP; Olson, 2024), a validated questionnaire that provides a measure of code-switching use/experience.

#### **Calculating successful prediction**

In most of our analyses, we collapsed across confidence levels (Maybe, Probably, Definitely judgments) and assessed participants’ sensitivity to upcoming code-switches with *d*-prime scores [z(*hits*) − z(*false alarms*)], which measure participants’ sensitivity to code-switches while accounting for response bias. We treated code-switches as the *signal*: “English” responses were categorized as *hits* when the upcoming word indeed was a code-switch, and as *false alarms* when the upcoming word did not switch. (Correspondingly, “Spanish” responses were *correct rejections* when the upcoming word was in Spanish and *misses* when the upcoming word switched to English, although note that correct rejections and misses are not incorporated into the d-prime scores.) “Extreme values” of hit or false alarm rates (0% or 100%) would yield infinite values of *d*-prime, so rates of zero were adjusted to 1/*n* and rates of one to 1 − 1/*n*, where *n* was the number of total trials (Macmillan & Kaplan, 1985). *D*-prime values above 0 indicate successful discrimination; values above 4 indicate near perfect discrimination.^2^

### Results

#### **Accuracy**

Participants achieved 54.73% accuracy on average (*SD* = 8.09%), with higher accuracy in the matched, no-switch condition (*M* = 76.48%, *SD* = 15.39%) than the code-switch condition (*M* = 37.50%, *SD* = 15.24%; see Table 2 for proportion of responses by condition). This pattern reflects the tendency to predict that an utterance starting in Spanish would continue in Spanish, which corresponds to real-world statistics of actual bilingual language use, where code-switches are relatively infrequent occurrences in bilingual speech (Beatty-Martínez et al., 2020; Fricke & Kootstra, 2016; Piccinini & Arvaniti, 2015). Using *d*-prime for our analysis (see below) allowed us to evaluate code-switch signal detection despite bilinguals’ reasonable bias to respond that sentences would stay in the same language.

**Table 2:** Experiment 1: Proportion of responses by condition

Code-switched to English		Stayed in Spanish	
Hit	Miss	Correct rejection	False alarm
0.21	0.35	0.34	0.10

#### **Code-switch prediction success**

**Fig. 2:**
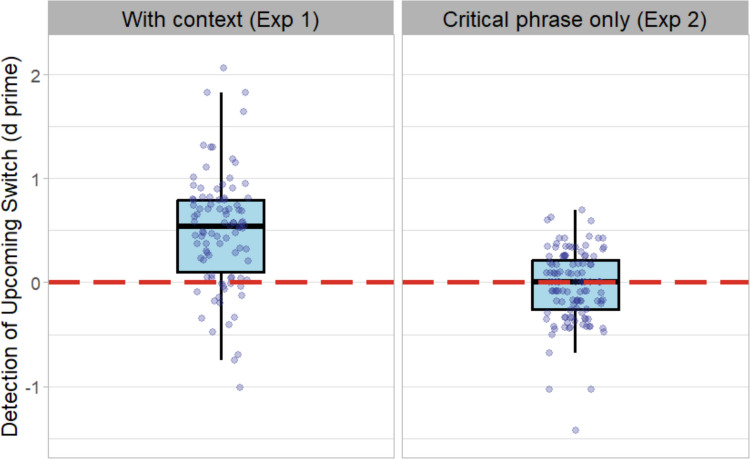
Experiment results. Each dot represents an individual’s *d*-prime score. (Color figure online)

Individuals’ *d*-prime values ranged from −1.01 to 2.06, with a mean of 0.49 (95% CI [0.38, 0.61]; see Fig. 2), which was significantly above zero according to a two-tailed *t* test^3^ (*t* = 8.82, *p* <.001). An exploratory one-sample Bayes factor analysis using a weakly informative prior (the “ttestBF” function’s default JZS prior with a scale of √2/2; Morey & Rouder, 2024) yielded a Bayes factor of 1.14e11, indicating that these data are much more likely to have occurred under the alternative hypothesis (mean ≠ 0) than the null hypothesis (mean = 0). Although they were far from perfect, the bilinguals were significantly above chance in predicting upcoming code-switches in ecologically valid input with prior context. Indeed, 77 of the 94 participants had *d*-prime values above 0.

#### **Individual factors in prediction success**

**Fig. 3:**
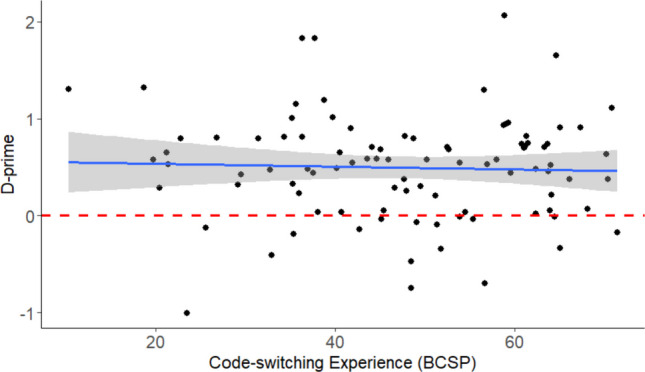
Experiment 1: *d*-prime values by code-switching experience. (Color figure online)

Against our prediction, individuals’ *d*-prime values did not correlate with code-switching experience (BCSP scores; *r* = −0.04, *p* =.71), as shown in Fig. 3. An exploratory Bayes factor correlation, run using the “correlationBF” function with the default Jeffreys-beta prior (Morey & Rouder, 2024), yielded a Bayes factor of 0.253, indicating that our data were somewhat more likely under the null hypothesis (*r* = 0) than the alternative hypothesis (*r* ≠ 0).

In exploratory analyses, individuals’ d-prime values also did not correlate with their ages of acquisition for each language, exposure entropy, LexTALE scores, pretask performance success prediction, or posttask performance evaluation (|*r*|s ≤.12, *p*s ≥.28). Additionally, *d*-prime scores did not correlate with participants’ averaged confidence ratings (rescoring “Definitely” responses as 3, “Probably” responses as 2, and “Maybe” responses as 1; *r* =.07, *p* =.51).

#### **Conversational context factors in prediction success**

We hypothesized that a conversation's characteristics may affect prediction success. Indeed, there were some differences between the two conditions in the 30 s of conversational context provided. Exploratory two-tailed *t* test analyses of the stimuli characteristics revealed that there was a nonsignificant trend for the code-switch condition’s conversations to have more overall prior code-switches (*M* = 5.83) than the no-switch condition’s conversations (*M* = 3.74; 95% CI [−0.14, 4.33]; *t* = 1.89, *p* =.07). Further, there were significantly more prior code-switches by the final-utterance speaker in the code-switch condition (*M* = 4.21) than in the no-switch condition (*M* = 2.42; 95% CI [0.19, 3.38]; *t* = 2.27, *p* =.03).

We additionally planned to evaluate how the following factors might influence prediction success: overall number of prior code-switches in that conversation, number of prior code-switches in that conversation specifically from the speaker who produced the final utterance, acoustic complexity (operationalized as compressed file size; Marin & Leder, 2013), and number of speakers in that conversation. In our preregistration, we planned to correlate these characteristics with the average *d*-prime scores for each item. However, it is impossible to calculate *d*-prime scores by item because the same item cannot have both hits and false alarms. Thus, we instead correlated these characteristics with average item accuracy, looking at code-switched and matched items separately. Despite the differences in stimuli characteristics across conditions reported above, none of the analyses revealed significant correlations between stimuli characteristics and prediction accuracy (|*r*|s ≤.39, *p*s ≥.10).

### Discussion

As hypothesized, bilinguals could predict code-switches in conversationally situated, ecologically valid speech. Their code-switch predictions were not perfect, but their above-chance code-switch detection is impressive, especially considering that they were listening to unfamiliar speakers that differed between trials, had only 30 seconds of conversational context, and had no visual cues. This finding suggests that in natural language comprehension, code-switches can be predicted, indicating that input language is predictable despite not affecting the conceptual message.

Against our hypothesis, there was no evidence that bilinguals with more code-switching experience were better at predicting code-switches. However, nearly all participants had some code-switching experience, which may have been enough to pick up on cues to an upcoming code-switch.

These successful code-switch predictions raise interesting questions for future work assessing what specific cues listeners might use to predict a code-switch. One possibility is that listeners capitalized on anticipatory articulation cues to make accurate predictions. Recall that VOT and speech rate can change before a code-switch occurs (Balukas & Koops, 2015; Fricke et al., 2016). The corpus from which we extracted our stimuli includes such pre-code-switch cues (Fricke et al., 2016); thus, these articulatory cues could guide listeners’ predictions.

Another (not mutually exclusive) possibility is that the bilinguals capitalized on the broader context provided by the 30 s of conversation to make accurate predictions. For example, listeners may have assumed speakers who code-switched more often in the 30 s of context would be more likely to code-switch in the critical utterance, although our analysis shows that prior code-switches did not clearly correlate with prediction success. However, participants may have used other contextual information to guide their predictions, such as speakers’ inferred ages and styles of speaking as well as the conversation’s setting and topic.

## Experiment 2

Although both articulatory and conversational factors might jointly contribute to bilinguals’ prediction success, an alternative hypothesis is that participants did not use the conversational context and instead relied solely on anticipatory articulation cues immediately prior to the audio cutoff. Under this hypothesis, bilinguals may not need additional context as the articulatory shifts naturally present in pre-code-switch production are sufficient to anticipate the switch. Since natural speech may include various articulatory cues (VOT, speech rate, phonotactic constraints, etc.), we opted to test this possibility in Experiment 2 by presenting only single clauses from single speakers—eliminating longer-range, conversational cues while preserving anticipatory articulation cues. If listeners’ successful predictions in Experiment 1 are based on pre-code-switch articulatory cues, then they should also accurately predict ecologically valid code-switches in these shorter contexts. Alternatively, if listeners rely on conversational context to make their predictions—or if larger context (or speaker familiarity) is important for the interpretation of those local articulatory cues—then they should be less able to predict code-switches from decontextualized short phrases. Based on prior work (Fricke et al., 2016), we hypothesized that bilinguals would successfully predict naturalistic code-switches even in short phrases and that bilinguals with more code-switching experience would be better predicters.

### Method

Experiment 2 followed the same method, except audio files included only the critical utterance with the final word cut off (Fig. 1). Thus, the audio files averaged 2.35 s in length (rather than being exactly 30 s, as in Experiment 1). Accordingly, the practice trials involved shorter audio files, and the instructions were modified slightly. Two additional differences were that an audio icon was not presented as the (much shorter) audio played, and the instructions did not specifically encourage quick responses.

Experiment 2 included 60 trials (30 code-switch and 30 matched). Here, we report results from all trials, but note that the pattern of results remains the same when only analyzing the 43 items matching those included in Experiment 1.

#### **Participants**

We recruited 120 US-based Spanish–English bilinguals through Prolific (*N* = 99) and the University participant pool (*N* = 21) with the same age and hearing requirements as in Experiment 1. We excluded five participants using the same preregistered exclusion criteria.

Our analysis included 115 bilinguals (58 women, 57 men). Participant characteristics in Experiment 2 largely mirrored those in Experiment 1 (Table 1)—demonstrating proficiency in both languages, young ages of acquisition for both languages, and generally more daily exposure to English (*M* = 71.83%) than Spanish (*M* = 26.95%). Most participants preferred using English (*N* = 80), although some preferred Spanish (*N* = 2) or had no preference (*N* = 33). The sample had varied code-switching experiences; ten participants reported never code-switching, but those who did started when they were young (*M* = 7.37 years, *SD* = 4.78).

### Results

#### Accuracy

Participants achieved 49.58% (*SD* = 5.79%) accuracy on average, with higher accuracy in the matched no-switch condition (*M* = 58.09%, *SD* = 11.14%) than the code-switch condition (*M* = 41.07%, *SD* = 13.52%; see Table 3 for proportion of responses by condition). This pattern again reflects the (reasonable) tendency to expect Spanish utterances to continue in Spanish. As before, we used d-prime to evaluate code-switch prediction because it is based on hits and false alarms for code-switches, circumventing the issue of participants’ bias to give no-switch responses.

**Table 3:** Experiment 2: Proportion of responses by condition

Code-switched to English		Stayed in Spanish	
Hit	Miss	Correct Rejection	False Alarm
0.21	0.29	0.29	0.21

#### **Code-switch prediction success**

Individuals’ d-prime values ranged from −1.43 to 0.69 with a mean of −0.04 (95% CI [−0.11, 0.02]; Fig. 2), which was not significantly different from zero according to a two-tailed *t* test (*t* = −1.29, *p* =.20). An exploratory one-sample Bayes factor analysis (again with the “ttestBF” function’s default JZS prior) yielded a Bayes factor of 0.231, making our data somewhat more likely under the null hypothesis (mean = 0) than the alternative hypothesis (mean ≠ 0). Thus, with longer-range conversational information removed, it seems that bilinguals could not discriminate upcoming code-switch versus no-switch utterances.

#### **Individual factors in prediction success**

**Fig. 4:**
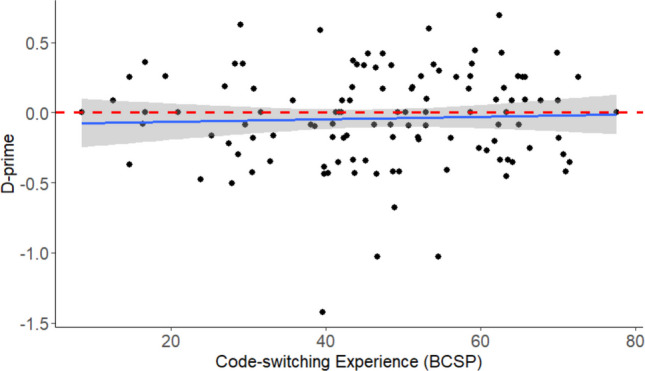
Experiment 2: *d*-prime values by code-switching experience. (Color figure online)

Against our prediction, individuals’ *d*-prime values did not correlate with their code-switching experience (BCSP scores; *r* =.04, *p* =.67; see Fig. 4). An exploratory Bayes factor correlation analysis (using the “correlationBF” function’s default Jeffreys-beta prior) yielded a Bayes factor of 0.234, indicating that our data were somewhat more likely under the null hypothesis (*r* = 0) than the alternative hypothesis (*r* ≠ 0).

Exploratory analyses also found no correlations between d-prime scores and individuals’ ages of acquisition for each language, exposure entropy, LexTALE scores, pretask performance success prediction, or posttask performance evaluation (|*r*|s ≤.11, *p*s ≥.25). However, an exploratory analysis found that d-prime scores correlated with participants’ averaged confidence ratings: Unexpectedly, participants who were overall more confident in their responses tended to have *lower d-*prime values (*r* = −.18, *p* <.05).

### Discussion

Without conversational context, bilinguals were unable to accurately predict code-switches, even in ecologically valid utterances that maintained anticipatory articulation cues. This suggests that bilinguals’ success in predicting code-switches in Experiment 1 was not driven solely by cues immediately preceding code-switches. Future research is needed to explore exactly which cues bilinguals do use to predict code-switches, but they may include conversational topic, speakers’ tendency to code-switch, and speakers’ demographics and social profile.

Of course, articulatory cues may still play a role in bilinguals’ ability to predict code-switches. For instance, bilinguals may rely on pre-switch articulatory cues to predict code-switches, but the ability to use those cues may require attunement to a speaker’s pronunciation patterns, explaining why prediction was successful in Experiment 1 but not Experiment 2. This account may also explain a contrast between our findings and prior work showing that articulatory cues can impact comprehension in short utterances. Unlike our experiment’s stimuli from multiple speakers, work finding impacts of pre-switch articulatory cues used stimuli from a single speaker (Fricke et al., 2016; Shen et al., 2020), potentially allowing listeners to become attuned to that individual’s production over many short utterances. Moreover, these phonetic cue studies may not actually show prediction per se but rather demonstrate that participants are worse at integrating an upcoming referent in the *absence* of naturalistic cues. Our Experiment 2 cannot specify exactly which cues listeners use to predict code-switches; however, it highlights that, even with ecologically valid stimuli, additional context is important in comprehension.

## General discussion

Bilinguals can consciously predict upcoming ecologically valid code-switches, but only with sufficient conversational context.

### What do comprehenders predict?

Bilinguals’ ability to successfully predict upcoming code-switches indicates that input language is a predictable feature for bilinguals. This finding strengthens evidence that comprehenders can predict at multiple levels of representation (e.g., Heilbron et al., 2022) and can inform our understanding of how predictive processes occur in comprehension. Even though code-switches do not fundamentally alter the sentence’s conceptual meaning relative to a single-language equivalent sentence, bilinguals could predict upcoming code-switches, suggesting that prediction is not restricted to attempting to infer the conceptual message (Kuperberg & Jaeger, 2016). Past work suggests that comprehenders are better-equipped to make specific, lexical-level predictions when the context is constrained (see Ryskin & Nieuwland, 2023, for a review) although most contemporary theories do not take into account code-switched language use. One possible explanation for our results is that the additional conversational context allowed listeners to narrow their expectations about upcoming content to successfully predict code-switches.

One important remaining question is whether bilingual comprehenders predict a language-specific lexical form or a broader language node. While this study cannot directly pinpoint the exact predictive process, prior work suggests that bilinguals do not experience lexical integration difficulties when encountering a high cloze code-switch—that is, when encountering a highly expected concept that was code-switched into another language (Moreno et al., 2002; Valdés Kroff et al., 2020). Taken together with the results presented here, the emerging picture is that bilinguals do not suffer from integration costs when encountering code-switches because they are anticipating when code-switches are most likely to occur (Valdés Kroff & Dussias, 2023). Admittedly, much more work in this nascent area is needed to fully understand bilingual sentence processing.

This research opens avenues for future work to address new questions about prediction in comprehension. While our data show that bilinguals *can* predict the language of upcoming speech, they cannot assess if bilinguals employ such predictions during real-time comprehension. Prediction theories remain divided on whether prediction is essential or optional (Ryskin & Nieuwland, 2023). Real-time prediction might also depend on individual or contextual factors. For example, the adaptive predictability hypothesis proposes that bilinguals may alter when they predict or not based on their cumulative code-switching experience—perhaps using articulatory cues to aid active prediction of upcoming code-switches and using morphosyntactic cues (such as patterns of when code-switches are likely based on prior exposure) to delay or suppress prediction when uncertainty (or the possibility of an upcoming code-switch) is high (Valdés Kroff & Dussias, 2023). Moreover, because code-switches are typically embedded in long stretches of unilingual language, adaptive prediction leads to holistic changes to how bilinguals handle prediction even during unilingual contexts.

Of course, our participants made explicit, untimed predictions, and so we cannot make claims about if/how they predict code-switches during online language processing. Nevertheless, this line of work adds to the body of literature that bilingual comprehension does indeed account for potential switches into another language without significant ‘costs’ to integration, bringing needed nuance to understanding prevalent lab-based switch costs. To address more specific questions on the exact mechanism of predictive processing in bilingual sentence processing, future work will need to incorporate real-time processing measures such as eye-tracking or EEG while bilinguals listen to contextualized, ecologically valid speech. Indeed, this study highlights the importance of considering ecological validity not only in code-switching research but in prediction research more generally, given our finding that prediction may differ based on how much context is provided. Further, future work should continue to explore the role of individual differences, despite our null effects of individual code-switching experience, given that prediction theories posit that individual language experience should affect predictive processing (e.g., Ryskin & Nieuwland, 2023; Valdés Kroff & Dussias, 2023).

### How did bilinguals predict upcoming code-switches?

Our results rule out the possibility that listeners’ predictions relied solely on decontextualized pre-code-switch articulatory cues; however, additional work is needed to specify which cues bilinguals use to predict input language and when these cues come online. Are articulatory cues the primary cues for language prediction but require speaker attunement? Or do conversational cues like setting or topic matter more? For example, perhaps listeners used the additional conversational context to evaluate the conversational topic and then predicted the language of the upcoming word based on their sociocultural associations of that topic. In general, it seems plausible that listeners may rely on different cues depending on the speaker, conversation, and their own language background. Indeed, in future work, it will be important to assess if our findings are generalizable to bilinguals of different backgrounds. For example, if code-switching experience is critical for predicting code-switches, then bilinguals who use their languages separately and are rarely exposed to code-switching may show different prediction patterns.

Additionally, our finding that naturalistic code-switches can be predicted suggests that code-switches may not be particularly costly to process when they align with natural language use. Prior work finding switch costs in comprehension may result in part from the unnaturally decontextualized presentation of code-switches. Future studies should test this hypothesis to determine if switch costs vary systematically based on the amount of conversational context provided.

In conclusion, this study establishes that listeners can predict the language of upcoming input. We also highlight the importance of considering ecological validity and context in psycholinguistic studies. Indeed, it may be difficult to guess what comes ____ if you don’t know what came before.

## Data Availability

The study materials and data for both experiments are available on OSF: https://osf.io/q9spv/

## Footnotes

1 One code-switched–matched-item pair did not have sufficient context preceding the code-switched utterance for Experiment 1; thus, Experiment 1 had 58 items and Experiment 2 had 60 items.

2 After data collection, we discovered errors for 15 trials in Experiment 1 where the audio did not end at the intended point. Those trials were excluded from analysis, leaving 43 critical trials (24 code-switched and 19 matched). For these 43 trials, the code-switched and matched trials did not differ in utterance word length (*t* = −0.06, *p* =.96) or critical word frequency (*t* = −0.26, *p* =.80). Note that the pattern of results was robust across multiple exclusion criteria: when analyzing the 43 included trials (results reported) or all 58 trials.

3 Although we preregistered one-tailed *t* tests, in this manuscript we report two-tailed analyses for both experiments to provide more informative confidence intervals. The pattern of results and significance does not change.

## Code availability

The R analysis scripts for both experiments are available on OSF: https://osf.io/q9spv/

## Declarations

### Open Practices Statement

Both experiments were preregistered (Experiment 1: https://osf.io/hf83m/; Experiment 2: https://osf.io/29uyc/). Materials, data, and analysis scripts are available on the Open Science Framework (OSF; https://osf.io/q9spv/).
